# Effect of daily mindfulness fluctuations on sleep and recovery-stress states in elite level judoka: an observational study

**DOI:** 10.3389/fspor.2025.1583058

**Published:** 2025-04-24

**Authors:** Tim Birnkraut, Michael Kellmann, Sarah Jakowski

**Affiliations:** ^1^ Faculty of Sport Science, Department of Sport Psychology, Ruhr University Bochum, Bochum, Germany; ^2^ School of Human Movement and Nutrition Sciences, The University of Queensland, St Lucia, QLD, Australia

**Keywords:** mindfulness, athletes, sleep, recovery, stress, monitoring

## Abstract

**Introduction:**

Sleep is a fundamental factor in an athlete's ability to sustain peak performance and endurance. Mindfulness, defined as a state of intentional, non-judgmental awareness of the present moment, has been linked to positive effects on sleep. The present study aims to investigate which recovery and sleep parameters are influenced by interindividual differences in mindfulness tendencies and intraindividual daily fluctuations in mindfulness.

**Methods:**

A two-week continuous monitoring study was conducted with 33 elite-level judoka (17 female, 16 male; age: *M* = 23.79, *SD* = 3.05) competing at the national and international level. Data collection included objective sleep monitoring via actigraphy and subjective monitoring through morning and evening self-report questionnaires. Sleep was analyzed as a function of both trait and daily mindfulness, as well as behavioral factors such as the number of training sessions, session intensity, and the implementation of recovery activities and naps.

**Results:**

Multilevel analyses revealed significant positive associations between mindfulness and qualitative subjective sleep parameters, as well as morning and evening recovery-stress states. Among the mindfulness facets, acting with awareness emerged as the strongest predictor. In terms of quantitative sleep parameters, mindfulness influenced both subjective and objective sleep latencies.

**Discussion:**

The findings suggest that mindfulness may play a key role in sleep regulation among athletes, particularly in enhancing perceived restfulness, improving recovery-stress states in the evening and morning, and facilitating the process of falling asleep. These results highlight mindfulness as a promising target for interventions aimed at improving subjective recovery and reducing sleep onset latencies through daily mindful behaviors. Furthermore, the study underscores the relative independence of qualitative and quantitative sleep parameters, suggesting they are influenced by distinct factors.

## 1 Introduction

Sleep plays a central role in an athlete's ability to not only adhere to demanding training schedules, but also to maximize the benefits of training by being able to deliver peak performance and endurance (1). Nevertheless, inadequate sleep quality and quantity are reported with above average frequency, especially in elite sport (2, 3). Common sleep problems include poor sleep quality, insufficient sleep duration, insomnia-related symptoms and daytime sleepiness (1). In competitive sports, there are several factors that can influence sleep at night and may contribute to the accumulation of sleep problems in athletes. On the one hand, these include sport factors such as high training loads, particularly early or late training or competition hours, or long journeys to competition venues, including unfamiliar sleep environments. On the other hand, there are non-sport factors such as social demands, work, study or family commitments or other individual characteristics that also affect non-athletic individuals (1, 4). Although it is still unclear which exact mechanisms are at work and what possibilities optimizing sleep behavior offers in terms of performance, it is widely acknowledged that sleep and recovery are highly relevant to performance for elite athletes and represent an important potential for improvement (5).

Judo is an Olympic martial art that demands exceptional physical and mental resilience (6). Competitive judo consists of high-intensity, intermittent actions that require a combination of strength, endurance, and coordination to achieve technical-tactical development and success in combat (7). During a match, athletes engage in dynamic physical exchanges, including gripping the opponent, disrupting their balance, and executing throwing techniques. These demands place significant strain on both the upper and lower body (8). As a weight-class sport, weight control plays a crucial role in competition preparation (9). Particularly in the period leading up to a competition, athletes often aim to reduce body mass within a short timeframe to gain a perceived advantage by competing against lighter and potentially weaker opponents (6, 10). The cumulative effects of intense, intermittent training loads, frequent weight fluctuations over the course of a season, and the necessity to peak at specific times present unique physiological and psychological challenges for elite judoka. These factors underscore the importance of recovery strategies such as sleep in maintaining performance levels (6, 11, 12). Consequently, systematic monitoring of stress and recovery is essential in the training process for judoka, aiding both athletes and coaches in optimizing training loads and recovery periods (5).

Sleep quality is a multifaceted construct that is challenging to define and measure objectively due to its subjective nature and individual variability (13). It can be assessed using both objective methods, such as polysomnography and actigraphy, and subjective approaches, including sleep diaries and psychometric questionnaires (14–16). While objective measures provide reliable data on sleep parameters, they may not fully capture subjective sleep experience, necessitating complementary self-report methods (17). Notably, sleep quality and sleep quantity appear to be relatively independent constructs (13). Objective parameters such as total sleep time (TST), sleep onset latency (SOL), and wake after sleep onset (WASO) often do not differ significantly between individuals with and without sleep complaints (13, 18). Moreover, while reduced sleep quantity is linked to clinical conditions such as depression, sleep quality is a stronger predictor of overall mental health and well-being in the general population (19–21).

Mindfulness is defined as a state of intentional, non-judgmental awareness of the present moment and has been shown to positively influence various health-related behaviors, including eating, sleeping, and substance use (22–24). Conceptually, mindfulness can be distinguished into two distinct constructs: Trait and state mindfulness (25, 26). While trait mindfulness refers to an individual's dispositional tendency to be mindful across different contexts, state mindfulness reflects momentary fluctuations in mindfulness during specific activities or situations (25, 26). Both trait and state mindfulness have been positively associated with sleep health (23, 27). However, these associations are primarily observed in subjective sleep parameters rather than objective sleep measures (28). Nonetheless, there is some evidence suggesting that mindfulness may positively influence objective sleep parameters, particularly by reducing sleep onset latencies (28, 29). Shallcross et al. (30) present an integrative etiological model of sleep disorders and elaborate on how mindfulness processes can influence the most important risk factors for sleep disorders. They describe five different cognitive and behavioral processes that contribute to triggering and maintaining sleep disorders. These include (a) excessive daytime and nighttime rumination, (b) primary arousal due to worry about the negative consequences of poor sleep, (c) secondary arousal due to negative metacognitive evaluation of primary arousal, (d) excessive monitoring and selective attention to internal or external sleep cues, combined with a dysfunctional need to control and increased sleep effort, and (e) distorted perceptions of sleep impairment. In terms of mindfulness, the authors distinguish three core processes of mindfulness: Experiential awareness, attention control and acceptance. They assume that increased awareness to internal and external experiences affects each of the five processes that contribute to the maintenance of sleep disorders. According to the model, attention control should be able to affect the first four of the processes mentioned. Finally, acceptance skills should promote a less conflictual and more flexible relationship with one's thoughts, emotions and sensations, and thus target the above listed processes (c) to (e). This model provides a good basis for explaining the positive effects of mindfulness on sleep quality that go beyond the development of sleep disorders. Mindfulness-based interventions have been shown to be effective in improving various biopsychosocial conditions such as depression, anxiety, stress, and insomnia (31–34). However, such interventions can also enhance well-being, athletic performance, and sleep quality in healthy individuals (35–38). Drawing clear causal conclusions regarding the specific parameters influenced by mindfulness and the potential benefit for elite athletes remains challenging. In particular, the effects of mindfulness-based interventions on sleep are less well established and robust than those on other psychological constructs (39, 40). A more comprehensive understanding of the conceptualization of mindfulness and its effects is necessary to optimize the effectiveness of mindfulness-based interventions in sports (41, 42).

Despite growing evidence that mindfulness is associated with improved sleep quality (23, 31), the underlying mechanisms remain insufficiently understood, particularly in elite sports settings. Most studies to date have focused on intervention-based designs, leaving a gap in understanding how natural daily fluctuations in mindfulness relate to sleep in real-world training environments. Addressing this gap, the present study investigates the influence of trait and daily mindfulness on subjective and objective sleep parameters in elite athletes. To this end, a two-week longitudinal monitoring study was conducted with elite judoka, assessing sleep parameters, trait and daily mindfulness, as well as behavioral factors such as training load and recovery activities. This within-person design was chosen because it allows for the examination of dynamic processes and temporally focused research questions (43, 44). Unlike cross-sectional approaches, within-person research enhances causal inferences about intrapersonal change (45, 46) and minimizes recall bias by using shorter assessment intervals that capture recent episodic experiences (44). Given the lack of a consistent definition and operationalization of the concept of sleep quality in research (13), the present study adopts a mixed-method approach, integrating objective (actigraphy) and subjective (sleep diaries) assessments of sleep quantity. Additionally, qualitative sleep parameters are examined by assessing perceived restfulness and recovery-stress states in the morning. A positive influence of mindfulness on these qualitative subjective parameters is hypothesized (H1). Building on the model proposed by Shallcross et al. (30) and other recent findings (47, 48), it is suggested that the effects of mindfulness on sleep are primarily related to the sleep onset phase. To examine this, the study will also assess evening recovery-stress states, with the assumption that mindfulness will have a beneficial effect on these states (H2). Furthermore, previous studies have investigated the impact of mindfulness on quantitative sleep parameters (28, 29), providing a basis for the hypothesis that increased mindfulness is associated with shorter sleep onset latencies (H3). Finally, a preliminary exploratory analysis will examine whether the effects of mindfulness differ between trait and daily mindfulness or whether specific facets of mindfulness exert differential effects on sleep parameters. Unlike experimental interventions, this study captures intraindividual variability in mindfulness and sleep as it naturally occurs within athletes' daily training environments. By reflecting real-world conditions, this research provides ecologically valid insights into self-regulation mechanisms that could inform future intervention development and recovery strategies in elite sports.

## 2 Materials and methods

### 2.1 Sample

The study was conducted in collaboration with the German Judo Federation. Data collection took place in two waves, aligning with men's and women's training camps. At the beginning of each camp, athletes were informed about the study's objectives and procedures and were given the opportunity to decide whether to participate. Exclusion criteria were the presence of a clinically diagnosed sleep disorder and being under the legal age of consent. The sample thus comprised athletes from the male and female Olympic squads, perspective squads, supplementary squads, and junior squads, including Olympic participants and medalists. According to the classification framework by McKay et al. (49), the participating athletes were classified as Level 4 (Elite/International Level) and Level 5 (World Class). A total of 33 athletes participated in the study (17 female, 16 male), with ages ranging from 20–33 years (*M* = 23.79, *SD* = 3.05). Six participants withdrew during the monitoring period. However, data collected up to the point of withdrawal were included in the analysis. Excluding these dropouts, compliance rates for subjective and objective monitoring procedures ranged from 78%–88%. In total, 328 nights of objective monitoring were analyzed, along with 379 evening protocols and 373 morning protocols.

### 2.2 Procedure

The study was conducted in two waves in September (for the male athletes) and October (for the female athletes) 2023. The study design consisted of a screening questionnaire to assess participants' trait mindfulness and demographic data followed by a two-week monitoring phase (including both subjective and objective assessments), with the first part taking place at a structured training camp and the second part occurring in individual home training environments. This resulted in five monitored nights in the training camp setting and nine monitored nights in individual training conditions. At the training camp, athletes followed a structured daily schedule with fixed time slots for meals (breakfast, lunch, and dinner) and organized training sessions. The day began with a morning activation session around 7:00 AM, followed by one to two judo-specific training sessions per day, primarily focusing on randori (a specific form of sparring). On some days, supplementary training sessions, such as strength or endurance training, were included. Additionally, dedicated recovery periods allowed athletes to engage in regeneration activities such as sauna or ice bath applications. While an official lights-out recommendation was set for 11:00 PM, individual nighttime routines and bedtimes were ultimately left to the athletes' discretion. In contrast, during the home training phase, each athlete's daily routine varied individually. Some athletes remained in their familiar training environments, while others traveled for national or international competitions. Whenever possible, and as long as they did not feel disrupted by the study procedures, athletes continued to participate in both subjective and objective monitoring during this phase. Prior to participation, all athletes provided written informed consent, and the study received ethical approval from the local ethics committee (Reference: EKS V 2023_14). The study was conducted in accordance with the principles of the Declaration of Helsinki.

### 2.3 Monitoring

Subjective monitoring was conducted to assess daily sleep patterns, training behaviors, and mindfulness. Sleep was evaluated using the short version of the evening-morning protocol (50). Based on participants' self-reported data, the following parameters were calculated: Subjective total sleep time (S-TST), subjective time in bed (S-TIB), subjective sleep onset latency (S-SOL), subjective sleep efficiency (S-SE), and subjective restfulness of sleep (S-RF; rated on a scale from 1 = very restful to 5 = not restful at all). The evening protocol was extended to include additional questions regarding the number of training sessions completed, recovery measures carried out, and the implementation and duration of daytime naps. The effects of naps of varying lengths remain a subject of ongoing research and appear to involve complex interactions. However, distinct types of naps have been identified. Short power naps of up to 30 min have been shown to be beneficial (51, 52), whereas other studies suggest that longer naps of approximately 90 min, which allow for a full sleep cycle, may be particularly advantageous (53–55). In contrast, medium-length naps of around 60 min have been associated with an increased risk of sleep inertia upon waking (53, 56, 57). Based on this evidence, naps were categorized into three groups: Power naps (P-naps; ≤30 min), complete sleep cycle naps (SC–Naps; 90–120 min), and other naps (O-naps; 31–89 min). Additionally, training session intensity was assessed using the Rating of Perceived Exertion Scale (RPE ranging from 0–10; 58). If multiple training sessions were completed in a single day, overall training intensity for that day was calculated as the mean of the reported RPE scores. Furthermore, a psychometric measure was included to assess daily mindfulness. Participants were instructed to complete the morning protocol before noon and the evening protocol after their final training session of the day but before bedtime.

Objective monitoring was carried out using the actigraphy decives SOMNOwatchTM plus (SOMNOmedics GmbH, Randersacker, Germany), which measure acceleration along three axes (*x*, *y*, *z*). This allows for the estimation of sleep-wake patterns based on movement intensity and frequency. Such activity monitors are a valid alternative to polysomnography for measuring the sleep of elite athletes (59). The actigraphy devices were worn by the participants at night on the wrist of the non-dominant arm. The participants were asked to mark the times of going to bed and getting up in the morning themselves using a push button on the device to set markers in the data. After recording, the raw actigraphic data was transferred to a computer and automatically analyzed using the DOMINO light software (version 1.5.0.11, SOMNOmedics GmbH, Randersacker, Germany). The *light on* and *light off* markers were manually set to match the markers set by the participants. The following six sleep parameters were calculated based on the actigraphy data: Objective sleep onset latency (O-SOL), objective total sleep time (O-TST), objective time in bed (O-TIB), objective wake after sleep onset (O-WASO), and objective sleep efficiency (O-SE).

### 2.4 Psychometric measures

#### 2.4.1 Screening

Trait Mindfulness was assessed using the German version of the Mindful Attention Awareness Scale (MAAS; 60), which is a 15-item questionnaire designed to ask participants to rate their ability to pay attention to the present moment and their awareness of everything experienced in the present moment on a scale from 1 = almost always to 6 = almost never. Higher scores indicate greater dispositional mindfulness. Like the original version developed by Brown and Ryan (61), the German version of the MAAS has a single-factor scale structure and shows high internal consistency (*α* = .80–.90), high test-retest reliability and good discriminant and convergent validity.

The German version of the Pittsburgh Sleep Quality Index (PSQI) was employed to assess sleep quality (62, 63). The PSQI is widely regarded as one of the most frequently used self-report instruments for evaluating sleep quality. It comprises 19 items and assesses seven clinically relevant domains of sleep disturbances: Subjective Sleep Quality, Sleep Latency, Sleep Duration, Sleep Efficiency, Sleep Disorders, Use of Sleep Medication, and Daytime Sleepiness. Each subscale is scored on a scale from 0–3. By summing the subscale scores, a global score reflecting overall sleep quality is obtained, ranging from 0–21. A global score exceeding 5 is generally indicative of poor sleep quality. The instrument demonstrates strong psychometric properties, including good internal consistency (*α* ≥ .80) and construct validity (62, 64), and has been validated as an effective measure of sleep quality (65).

#### 2.4.2 Daily measures

Daily Mindfulness was assessed by the Multidimensional State Mindfulness Questionnaire (MSMQ; 66). The MSMQ consists of the three scales acting with awareness (MSMQ-1), non-judgmental acceptance (MSMQ-2), and present-moment attention (MSMQ-3), which reflect closely the aspects of mindfulness described in the model by Shallcross et al. (30). Each scale consists of three items and thus enables an economic assessment of state mindfulness with a total of only nine items. The instructions were adapted to the presentation in the evening protocol as follows: “Please think back to today: How did you behave, what was going on inside you? The following questions relate to this”. The items are each to be answered on a scale from 0 = does not apply at all to 6 = applies strongly. Higher scores on all three subscales indicate greater mindfulness, with higher values on MSMQ-1 reflecting increased acting with awareness, higher values on MSMQ-2 indicating greater non-judgmental acceptance, and higher values on MSMQ-3 representing stronger present-moment attention. The authors report reliabilities between *α* = .63 and *α* = .74 for the three scales (67). Correctly conceptualizing, calculating, and interpreting within-person reliability is one of the challenges of within-person research. Since user-friendly analysis tools for calculating within-person reliability in classical statistical programs are currently still lacking, the software provided by Yang et al. (44) for calculating within-person reliability is used for the level-1 questionnaires. The calculated coefficient is an extension of the classical Cronbach's alpha, proposed by Geldhof et al. (68). It adapts the Cronbach's alpha formula by using level-specific variance components to calculate between-person alpha and within-person alpha. It is therefore suitable for multilevel research and for within-person research. There is also evidence that the interpretation of within-person reliability allows for deviations from the interpretation of between-person reliability (44, 69). Following the recommendations of Yang et al. (44), a threshold of 0.7 is applied for a scale length of at least three items. For the present sample the within-person reliabilities are *α* = .81 for the MSMQ-1, *α* = .70 for the MSMQ-2, and *α* = .77 for the MSMQ-3. The values are therefore all within an acceptable range.

The current recovery-stress state was assessed using the German version of the Short Recovery and Stress Scale (SRSS; 70, 71). This scale provides an efficient, multidimensional measure of recovery-stress states across emotional, mental, physical, and general domains using eight items. The SRSS was developed as a condensed version of the Acute Recovery and Stress Scale (ARSS; 70). SRSS items are named after the corresponding ARSS scales and are rated on a seven-point Likert scale (0 = does not apply at all, 6 = fully applies). The first four items assess the recovery dimension (Physical Performance Capability, Mental Performance Capability, Emotional Balance, Overall Recovery), while the last four measure the stress dimension (Muscular Stress, Lack of Activation, Negative Emotional State, Overall Stress). Higher scores indicate greater levels of recovery or stress in the respective domains. Due to considerable interindividual variability, SRSS scores should always be interpreted in relation to intraindividual changes through repeated measurements. The scale demonstrates strong construct validity and high sensitivity to change, including the relation to sleep quality parameters (70, 72).

### 2.5 Data analysis

Data analysis was conducted in IBM SPSS v.29. First, descriptive statistics were calculated for the screening and monitoring variables. To examine the relationships between mindfulness and sleep parameters as well as recovery-stress states in the morning and evening, multilevel linear models were applied. In the models, Level 1 represented intraindividual differences, allowing for the examination of variations in sleep quality within individuals across multiple nights. Level 2 captured interindividual differences, assessing whether variations in sleep quality were attributable to differences between athletes. To account for the repeated-measures design, random intercepts were included to represent individual variability. The significance threshold was set at *p* < .05, with a 95% confidence interval for all estimates. Parameter estimation was performed using a restricted maximum likelihood approach with Kenward-Roger approximation (73). Daily mindfulness (MSMQ-1, MSMQ-2, MSMQ-3) and behavioral factors such as the number of training sessions, perceived training intensity, and engagement in recovery activities were included as level-1 predictors. Trait mindfulness (MAAS) was incorporated as a level-2 predictor. Control variables included gender, training camp environment (TC-E), and the implementation of daytime naps. Naps were categorized as power naps (P-Nap: up to 30 min), full sleep cycle naps (SC-Nap: 90 min or more), and other naps (O-Nap: 31–89 min). A total of 26 models were calculated: Five based on objective sleep quantity parameters (O-TIB, O-TST, O-SE, O-SOL, O-WASO), four on subjective sleep quantity parameters (S-TIB, S-TST, S-SE, S-SOL), eight on evening recovery-stress states assessed (SRSS), and nine on morning recovery-stress states and subjective sleep quality parameters (SRSS, S-RF). All level-1 variables were centered around the individuals' mean to distinguish within-person effects from between-person effects (44). The level-2 predictor (MAAS) was centered around the grand mean to enhance interpretability of the coefficients. Binary control variables were not centered.

## 3 Results

### 3.1 Descriptive statistics

Descriptive statistics for the screening and monitoring variables are presented in Table 1. The average PSQI score in the sample exceeded the clinical cut-off. Specifically, 16 out of 33 athletes fell into the unfavorable range, including 10 male athletes. Over the course of the monitoring period, a total of 123 naps were recorded, comprising 58 P-naps, 21 SC-naps, and 44 O-naps. Training sessions were conducted on 286 of the 402 days assessed, with 134 days featuring one session, 132 days featuring two sessions, and 20 days featuring three sessions. The average perceived session intensity was *M* = 4.74 (*SD* = 1.86). Systematic recovery measures were implemented on 109 days, with 73 days involving one recovery activity, 27 days involving two, seven days involving three, and two days involving four recovery activities. The most frequently reported recovery measures included massages and physiotherapy, ice baths or cold therapy, and sauna sessions. To contextualize the sleep parameters observed in this sample, the sleep quality recommendations of the National Sleep Foundation (74, 75) provide a useful reference. Among the objective parameters, the average O-SE and O-SOL fell within a favorable range. However, O-WASO was markedly elevated, exceeding 50 min—a threshold generally considered indicative of poor sleep quality. The average O-TST was at the lower end of the recommended 7–9 h for this age group. In terms of subjective parameters, athletes reported longer S-TST, S-TIB, and S-SOL compared to their corresponding objective measurements. The calculated S-SE, derived from self-reported total sleep time and time in bed, was substantially higher than its objective counterpart. In some cases, subjective estimates even exceeded 100%, highlighting potential biases in athletes' sleep perception. Gender differences can also be descriptively observed. Female athletes reported more favorable values for nearly all subjective parameters, except for S-SOL. Regarding objective measures, female athletes had longer average O-TST, O-TIB, and O-WASO compared to their male counterparts.

**Table 1:** Descriptive statistics for screening and monitoring variables.

Variable	Total (*N* = 33)	Female (*n = 17)*	Male (*n* = 16)
MAAS [0–21]	3.81 ± 0.68	3.75 ± 0.70	3.88 ± 0.67
PSQI [1–6]	5.94 ± 2.51	6.06 ± 1.43	5.81 ± 3.35
S-TST (min)	474.0 ± 80.1	492.9 ± 70.9	452.9 ± 84.5
S-TIB (min)	524.1 ± 72.2	536.0 ± 72.6	509.7 ± 69.1
S-SOL (min)	17.2 ± 22.3	19.7 ± 23.7	14.5 ± 20.3
S-SE (%)	91.4 ± 11.2	92.5 ± 12.5	90.1 ± 9.2
S-RF [1–5]	2.4 ± 0.9	2.2 ± 0.9	2.5 ± 0.8
O-TST (min)	420.3 ± 68.5	439.8 ± 63.4	387.7 ± 64.4
O-TIB (min)	510.8 ± 74.4	534.8 ± 67.8	470.8 ± 67.8
O-SOL (min)	10.2 ± 12.1	9.7 ± 12.2	11.1 ± 11.9
O-WASO (min)	80.3 ± 39.3	85.3 ± 43.4	72.1 ± 29.8
O-SE (%)	82.4 ± 6.9	82.4 ± 7.3	82.3 ± 6.2

Notes: Interindividual means and standard deviations of subjective and objective sleep parameters. MAAS = mindful attention awareness scale (higher scores indicate greater mindfulness); PSQI = Pittsburgh sleep quality index (lower scores indicate better sleep quality); S-TST = subjective total sleep time; S-TIB = subjective time in bed; S-SOL = subjective sleep onset latency; S-SE = subjective sleep efficiency; S-RF = subjective restfulness of sleep (from 1 = very restful to 5 = not restful at all); O-TST = objective total sleep time; O-TIB = objective time in bed; O-SOL = objective sleep onset latency; O-WASO = objective wake after sleep onset; O-SE = objective sleep efficiency.

### 3.2 Multilevel analyses

Tables 2–4 show the significant parameters of the multilevel analysis sorted by dependent variable. The complete set of parameters for each model can be found in the supplementary material.

**Table 2:** Significant parameters of the multilevel analyses for quantitative sleep parameters sorted by dependent variable.

Outcome	*B*	*SE*	*F*	(df)	*p*
O-TIB					
RPE	−7.635	2.598	8.636	(1, 199.931)	.004
Gender	56.212	10.371	29.380	(1, 27.103)	<.001
O-TST					
RPE	−6.880	2.207	9.723	(1, 193,029)	.002
P-Nap	−26.943	8.657	9.687	(1, 209,721)	.002
Gender	46.035	14.096	10.666	(1, 27,906)	.003
O-SE					
Sessions	−1.526	.684	4.973	(1, 204,723)	.027
Recovery	1.323	.556	5.667	(1, 205,383)	.018
P-Nap	−2.963	.963	9.461	(1, 210,981)	.002
O-SOL					
Sessions	4.185	1.363	9.427	(1, 202,399)	.002
MAAS	−3.920	1.740	5.075	(1, 20,599)	.035
SC-Nap	11.671	3.349	12.148	(1, 207,085)	<.001
O-WASO					
MSMQ-1	−5.430	2.643	4.221	(1, 174,085)	.041
P-Nap	13.125	5.747	5.216	(1, 207,357)	.023
S-TIB					
RPE	−8.862	2.904	9.310	(1, 226.859)	.003
Gender	35.612	9.658	13.596	(1, 27.947)	<.001
S-TST					
MSMQ-1	12.818	5.125	6.256	(1, 175.459)	.013
RPE	−9.631	2.820	11.661	(1, 213.854)	<.001
P-Nap	−25.829	11.059	5.455	(1, 252.294)	.020
O-Nap	−28.312	13.152	4.634	(1, 248.902)	.032
Gender	37.396	10.637	12.360	(1, 24.264)	.002
S-SOL					
MSMQ-3	−5.580	2.379	5.501	(1, 235.259)	.020
Recovery	−4.814	2.241	4.614	(1, 230.736)	.033
O-Nap	13.688	4.469	9.380	(1, 243.053)	.002

Notes: Dependent variables: O-TIB = objective time in bed; O-TST = objective total sleep time; O-SE = objective sleep efficiency; O-SOL = objective sleep onset latency; O-WASO = objective wake after sleep onset; S-TIB = subjective time in bed; S-TST = subjective total sleep time; S-SE = subjective sleep efficiency; S-SOL = subjective sleep onset latency; Independent variables: MSMQ-1 = Acting with Awareness, MSMQ-2 = Non-judgmental Acceptance, MSMQ-3 = Present-moment Attention, Sessions = number of training sessions on the previous day, RPE = average intensity of the training sessions, MAAS = Mindful Attention Awareness Scale, P-Nap = completion of a power nap on the previous day (binary), O-Nap = completion of another nap on the previous day (binary); SC-Nap = completion of full sleep cycle nap an the previous day (binary); Gender = gender of the participant (binary: 0 = male, 1 = female).

**Table 3:** Significant parameters of the multilevel analyses for qualitative morning parameters.

Outcome	*B*	*SE*	*F*	(df)	*p*
S-RF					
MSMQ-1	−0.209	0.062	11.499	(1, 221.744)	<.001
RPE	0.068	0.033	4.330	(1, 235.514)	.039
MAAS	−0.198	0.079	6.236	(1, 24.784)	.020
P-Nap	0.468	0.123	14.538	(1, 231.611)	<.001
O-Nap	0.346	0.145	5.681	(1, 226.175)	.018
Gender	−0.334	0.111	9.051	(1, 27.607)	.006
Morning Physical Performance Capability					
MSMQ-1	0.188	0.074	6.466	(1, 205.263)	.012
Sessions	−0.232	0.106	4.844	(1, 227.395)	.029
RPE	−0.135	0.040	11.673	(1, 230.780)	<.001
TC-E	−0.545	0.143	14.482	(1, 108.106)	<.001
Morning Mental Performance Capability					
MSMQ-1	0.170	0.082	4.322	(1, 193.527)	.039
Sessions	−0.364	0.119	9.360	(1, 230.045)	.002
RPE	−0.150	0.044	11.491	(1, 226.288)	<.001
P-Nap	−0.409	0.177	5.321	(1, 237.868)	.022
TC-E	−0.521	0.154	11.393	(1, 114.926)	.001
Morning Emotional Balance					
Sessions	−0.326	0.115	8.027	(1, 230.172)	.005
RPE	−0.116	0.043	7.314	(1, 228.100)	.007
P-Nap	−0.375	0.171	4.804	(1, 233.731)	.029
TC-E	−0.331	0.151	4.792	(1, 112.052)	.031
Morning Overall Recovery					
MSMQ-1	0.200	0.084	5.650	(1, 218.030)	.018
Sessions	−0.348	0.118	8.687	(1, 224.523)	.004
RPE	−0.233	0.045	27.358	(1, 232.113)	<.001
TC-E	−0.376	0.167	5.075	(1, 117.245)	.026
Morning Muscular Stress					
Sessions	0.519	0.118	19.508	(1, 214.194)	<.001
RPE	0.210	0.045	21.947	(1, 228.346)	<.001
O-Nap	0.445	0.200	4.934	(1, 199.557)	.027
Morning Lack of Activation					
MSMQ-1	−0.190	0.091	4.350	(1, 197.482)	.038
Sessions	0.269	0.130	4.261	(1, 229.325)	.040
RPE	0.115	0.049	5.553	(1, 228.914)	.019
TC-E	0.575	0.175	10.781	(1, 104.978)	.001
Morning Negative Emotional State					
P-Nap	0.436	0.168	6.734	(1, 209.317)	.010
Morning Overall Stress					
Sessions	0.419	0.112	13.959	(1, 205.733)	<.001
RPE	0.230	0.043	28.310	(1, 224.760)	<.001
MAAS	−0.357	0.150	5.697	(1, 26.670)	.024
O-Nap	0.423	0.188	5.083	(1, 192.168)	.025

Notes: Dependent variables: S-RF = subjective restfulness of sleep (1 = very restful to 5 = not restful at all); Independent variables: MSMQ-1 = Acting with Awareness, Sessions = number of training sessions on the previous day, RPE = average intensity of the training sessions, MAAS = Mindful Attention Awareness Scale, P-Nap = completion of a power nap on the previous day (binary), O-Nap = completion of another nap on the previous day (binary); TC-E = training camp environment (binary: 0 = home training, 1 = training camp).

**Table 4:** Significant parameters of the multilevel analyses for qualitative evening parameters.

Outcome	*B*	*SE*	*F*	(df)	*p*
Evening Physical Performance Capability					
MSMQ-1	0.268	0.079	11.505	(1, 245.373)	<.001
TC-E	−0.363	0.182	3.974	(1, 123.690)	.048
Evening Mental Performance Capability					
MSMQ-1	0.243	0.080	9.196	(1, 233.009)	.003
MSMQ-3	0.217	0.106	4.186	(1, 244.781)	.042
Recovery	0.206	0.099	4.312	(1, 238.726)	.039
TC-E	−0.395	0.163	5.907	(1, 122.260)	.017
Evening Emotional Balance					
MSMQ-2	0.202	0.073	7.673	(1, 245.407)	.006
Evening Overall Recovery					
MSMQ-1	0.375	0.084	20.102	(1, 248.165)	<.001
Sessions	−0.343	0.120	8.152	(1, 237.889)	.005
RPE	−0.142	0.045	10.074	(1, 246.782)	.002
TC-E	−0.460	0.180	6.572	(1, 132.160)	.011
Evening Muscular Stress					
MSMQ-1	−0.223	0.087	6.487	(1, 244.829)	.011
Sessions	0.285	0.123	5.383	(1, 223.332)	.021
RPE	0.181	0.046	15.179	(1, 237.554)	<.001
TC-E	0.473	0.207	5.213	(1, 134.654)	.024
Evening Lack of Activation					
TC-E	0.460	0.162	8.099	(1, 127.393)	.005
Evening Negative Emotional State					
MSMQ-2	−0.293	0.089	10.961	(1, 240.821)	.001
O-Nap	−0.517	0.230	5.039	(1, 242.814)	.026
Evening Overall Stress					
MSMQ-1	−0.297	0.093	10.177	(1, 248.947)	.002
Sessions	0.396	0.133	8.905	(1, 231.917)	.003
RPE	0.196	0.050	15.564	(1, 244.058)	<.001

Notes: Dependent variables: S-RF = subjective restfulness of sleep (1 = very restful to 5 = not restful at all); Independent variables: MSMQ-1 = Acting with Awareness, MSMQ-2 = Non-judgmental Acceptance, MSMQ-3 = Present-moment Attention, Sessions = number of training sessions on the previous day, RPE = average intensity of the training sessions, O-Nap = completion of another nap on the previous day (binary); TC-E = training camp environment (binary: 0 = home training, 1 = training camp).

#### 3.2.1 Results for quantitative sleep parameters

Intraindividually, MSMQ-1 was associated with shorter O-WASO (*p* = .041) and longer S-TST (*p* = .013). Higher MSMQ-3 was associated with shorter S-SOL (*p* = .020). Regarding behavioral predictors, higher number of sessions was associated with lower O-SE (*p* = .027) and longer O-SOL (*p* = .002), and higher training intensity was associated with shorter O-TIB (*p* = .004), O-TST (*p* = .002), S-TIB (*p* = .003), and S-TST (*p* < .001). In contrast, performing systematic recovery activities predicted higher O-SE (*p* = .018) and shorter S-SOL (*p* = .033).

At the interindividual level, higher MAAS scores predicted shorter O-SOL (*p* = .035). In addition, female gender was a predictor of longer objective and subjective TIB (objective: *p* < .001; subjective: *p* < .001) and TST (objective: *p* = .003; subjective: *p* = .002). P-naps predicted shorter O-TST (*p* = .002) and S-TST (*p* = .020), reduced O-SE (*p* = .002), and prolonged O-WASO (*p* = .023) in the following night. SC-naps were associated with prolonged O-SOL (*p* < .001). O-naps were also a predictor of shorter S-TST (*p* = .032) and prolonged S-SOL (*p* = .002).

#### 3.2.2 Results for subjective morning variables

Within individuals, higher MSMQ-1 predicted higher morning scores for Physical Performance Capability (*p* = .012), Mental Performance Capability (*p* = .039), and Overall Recovery (*p* = .018), lower scores for morning Lack of Activation (*p* = .038), as well as increased subjective restfulness (*p* < .001). Regarding behavioral factors, a higher number of training sessions was associated with decreased scores on the recovery scales (*p* = .029; *p* = .002; *p* = .005; *p* = .004) and increased scores on Muscular Stress (*p* < .001), Lack of Activation (*p* = .040), and Overall Stress (*p* < .001) in the morning. Exercise intensity was also associated with decreased morning scores on the recovery scales (*p* < .001; *p* < .001; *p* = .007; *p* < .001) and increased scores on Muscular Stress (*p* < .001), Lack of Activation (*p* = .019), and Overall Stress (*p* < .001). In addition, higher intensity also predicted lower subjective restfulness of sleep (*p* = .039).

Interindividually, higher MAAS scores predicted higher subjective restfulness of sleep (*p* = .020) and lower morning Overall Stress scores (*p* = .024). In addition, taking P-naps was associated with decreased subjective restfulness of sleep (*p* < .001), decreased Mental Performance Capability (*p* = .022) and Emotional Balance (*p* = .029), and increased morning Negative Emotional State (*p* = .010). Taking an O-nap was also associated with decreased restfulness (*p* = .018) and increased Negative Emotional State (*p* = .027) and Overall Stress (*p* = .025) morning scores.

Female gender was a predictor of higher subjective sleep restfulness (*p* = .006). Finally, time spent at training camp vs. time spent training at home was a predictor of lower morning scores for Physical Performance Capability (*p* < .001), Mental Performance Capability (*p* = .001), Emotional Balance (*p* = .031), and Overall Recovery (*p* = .026), and higher scores for Lack of Activation (*p* = .001).

#### 3.2.3 Results for subjective evening variables

Intraindividually, higher MSMQ-1 predicted higher evening scores on Physical Performance Capability (*p* < .001), Mental Performance Capability (*p* = .003), and Overall Recovery (*p* < .001), and predicted lower evening scores on Lack of Activation (*p* = .011) and Overall Stress (*p* = .002). Higher MSMQ-2 was associated with higher Emotional Balance scores (*p* = .006) and decreased Negative Emotional State scores (*p* = .001) in the evening. MSMQ-3 was a positive predictor of higher Mental Performance Capability evening scores (*p* = .042). At the behavioral level, a higher number of training sessions predicted decreased Overall Recovery scores (*p* = .005) and increased scores for Muscular Stress (*p* = .021) and Overall Stress (*p* = .003) the following evening. In addition, training intensity was a predictor of decreased Overall Recovery scores (*p* = .002) and increased scores for Muscular Stress (*p* < .001) and Overall Stress (*p* < .001). The implementation of systematic recovery activities, on the other hand, was associated with increased Mental Performance Capability scores (*p* = .039) in the evening.

When considering the influence of daytime napping, only taking O-naps was associated with decreased Negative Emotional State scores (*p* = .026) in the evening. Consistent with the results regarding morning variables, training camp environment was a predictor of decreased Physical Performance Capability scores (*p* = .048), decreased Mental Performance Capability scores (*p* = .017), decreased Overall Recovery scores (*p* = .011), increased Muscular Stress scores (*p* = .024), and increased Lack of Activation scores (*p* = .005) in the evening.

## 4 Discussion

The present monitoring study aimed to investigate the influence of both trait and daily mindfulness on sleep in elite athletes. In addition to mindfulness, behavioral variables such as training load, training intensity, and the implementation of systematic recovery strategies or daytime naps were considered. Using multilevel modeling, the study differentiated between interindividual and intraindividual effects on subjectively and objectively measured quantitative and qualitative sleep parameters. The results suggest that both daily and trait mindfulness have a positive impact on qualitative sleep parameters. Moreover, all three facets of daily mindfulness were positively associated with evening recovery-stress states, suggesting a beneficial impact on the pre-sleep phase. Additionally, higher daily mindfulness was linked to shorter subjective sleep onset latency, while higher trait mindfulness was associated with shorter objective sleep onset latency.

As anticipated, mindfulness demonstrated beneficial predictive effects on sleep and recovery-stress states (H1), aligning with current research findings (23, 76, 77). Both trait mindfulness and daily mindfulness predicted higher subjective sleep quality. On an interindividual level, the results further suggest that athletes with higher trait mindfulness experience greater subjective sleep restfulness and report more favorable morning stress states. These findings are consistent with existing literature, which also indicates a positive association between higher mindfulness levels and sleep quality (27, 78, 79). On an intraindividual level, only the mindfulness facet acting with awareness was associated with qualitative sleep parameters assessed in the morning. Specifically, higher levels of acting with awareness were primarily linked to improved perceived recovery. Conceptually, acting with awareness reflects the extent to which an individual is inattentive to present activities and is therefore closely related to present-moment attention. However, acting with awareness is assessed more implicitly, capturing inattentiveness in daily activities through negatively formulated items (67). Blanke and Brose (66) reported that individuals experienced fewer negative and more positive emotions during periods of heightened acting with awareness, non-judgmental acceptance, and present-moment attention. However, the facet acting with awareness did not demonstrate predictive value beyond the other two facets. The authors concluded that present-moment attention and non-judgmental acceptance may sufficiently explain affective well-being and suggested a two-factor model. This contrasts with the present findings, which indicate that acting with awareness plays a central role in the relationship between mindfulness and sleep quality. Moreover, other studies support these results. For instance, Lau et al. (80) reported that awareness and acceptance are key mechanisms in mindfulness interventions for improving sleep quality, partly by reducing psychological stress. Additionally, the model proposed by Shallcross et al. (30), which outlines various mindfulness-related mechanisms in the development and maintenance of sleep disorders, assigns experiential awareness a crucial role, as it is the only mindfulness component thought to influence all cognitive and behavioral processes associated with sleep disturbances. Similarly, Sala et al. (79) concluded in their meta-analysis that acting with awareness was the most consistent predictor of health behaviors, including sleep.

Regarding the results for evening qualitative parameters (H2), it is noticeable that while daily mindfulness is associated with evening recovery-stress states, trait mindfulness is not. Once again, a predictive effect is particularly evident for the facet acting with awareness, which in this context is linked to both more favorable recovery states and lower stress levels. Notably, higher daily acting with awareness also serves as a beneficial predictor for explicitly physical parameters, such as Physical Performance Capability and Muscular Stress. Other studies have similarly demonstrated that mindfulness can have effects on the physical level and that mindfulness-based approaches may be effectively utilized in physical rehabilitation (81–83). Taken together, these findings support the hypothesis that one mechanism underlying the positive effects of mindfulness on sleep quality is its influence on the phase preceding sleep onset. Future research should further explore potential mediator variables in this relationship. For instance, Smith et al. (28) describe a mechanism in which mindfulness affects sleep via reductions in rumination and negative affect. Similarly, Pawsey et al. (77) report that daily fluctuations in mindfulness influence sleep quality, with rumination acting as a mediator. Other studies, however, have identified perceived stress or cognitive pre-sleep arousal as mediating factors (48, 78).

Furthermore, the results indicate a beneficial influence of mindfulness on both subjective and objective sleep latencies (H3). Specifically, higher trait mindfulness predicts shorter objective sleep latencies, while at the intraindividual level, greater present-moment attention is associated with shorter subjective sleep latencies. These findings build upon previous evidence suggesting that mindfulness positively affects quantitative sleep parameters, particularly sleep latency (28, 29). It is worth noting that these effects are of a similar magnitude to those of behavioral factors, such as the number of training sessions or the implementation of systematic recovery activities. Moreover, both subjective and objective sleep latencies in the present sample already fall within a highly favorable range (74, 75). It can be assumed that the beneficial effects of mindfulness on sleep latencies may be even more pronounced in individuals with initially poorer sleep parameters (32), a phenomenon also observed in interventions targeting affective disorders such as depression (84, 85).

In summary, both trait and daily mindfulness demonstrate beneficial effects on qualitative and quantitative sleep parameters, with the results largely supporting the proposed hypotheses. Within the construct of mindfulness, a nuanced pattern emerges: At the interindividual level, trait mindfulness is associated with greater subjective restfulness of sleep and shorter objective sleep onset latencies. At the intraindividual level, the results indicate that the facet of acting with awareness serves as a particularly strong predictor of beneficial recovery-stress states in the evening, as well as improved recovery values the following morning. Additionally, present-moment attention predicts shorter subjective sleep latencies. These findings suggest that integrating mindfulness interventions into an athlete's routine could be highly beneficial. The strong associations between daily mindfulness and, in particular, qualitative sleep parameters have several practical implications for the development and implementation of such interventions. Notably, mindfulness training should incorporate informal practice (77, 86, 87). Informal practice integrates mindfulness into everyday activities and existing routines, such as mindful eating, mindful household tasks, or other mindful moments. Even a small, acute increase in daily mindfulness can have a positive impact on well-being (77). For instance, Li et al. (88) report that short mindfulness exercises can reduce arousal before bedtime, enhance recovery, and improve overall sleep quality. Digital mindfulness training may represent an effective method for reaching athletes in their daily lives. This delivery format ensures accessibility to mindfulness exercises, and even brief interventions have demonstrated positive effects (77, 89, 90).

The analysis of sport-related behavioral variables reveals unfavorable effects of both the number of training sessions and session intensity on quantitative and qualitative sleep parameters. Specifically, higher session intensity predicts shorter subjective and objective TST and TIB, while an increased number of training sessions negatively impacts objective SE and SOL. Existing literature also indicates that high training loads and the associated mental strain can impair sleep quality (4, 91). Regarding qualitative sleep parameters, both the frequency and intensity of training sessions were associated with lower scores on recovery scales and higher scores on stress scales in the evening and the following morning, an effect that appears intuitively plausible. A buffering effect of systematic recovery measures was identified for objective SE, subjective sleep SOL, and evening Mental Performance Capability. One possible reason why no further beneficial effects of recovery measures on qualitative sleep parameters were observed is that most of these activities (approximately 70%) took place during the training camp, a period in which athletes generally exhibited less favorable recovery-stress states in the evening and morning.

Napping represents a behavioral variable not directly related to sports. The question of whether naps are beneficial for athletes remains a complex and debated topic in current research. Numerous studies report potential positive effects of naps on daytime sleepiness and physical performance without negatively impacting nighttime sleep quality (92–95). However, some findings suggest otherwise. For example, Petit et al. (96) found that daytime naps not only lack reliable short-term performance benefits for athletes but may also negatively affect nighttime sleep, particularly by prolonging sleep onset latencies. Based on current literature, short P-naps and substantially longer SC-naps are generally considered beneficial, while O-naps pose a greater risk of sleep inertia (51, 55, 56). The present results indicate unfavorable effects of P-naps and O-naps, particularly in relation to reduced subjective and objective sleep time, decreased sleep efficiency, prolonged sleep onset latency, and increased wake time after sleep onset. Additionally, the subjective restfulness of nighttime sleep was markedly lower when P-naps and O-naps were taken. In contrast, SC-naps were associated with longer objective sleep latencies but did not negatively impact other qualitative or quantitative sleep parameters. These findings align with current recommendations advocating for naps lasting at least 90 min (53–55). It should be noted that the present study did not account for the timing of naps during the day, which may be an important influencing factor (93).

Finally, gender was included as a control variable in the present study. Female gender emerged as a predictor of both objectively and subjectively longer TIB and TST, as well as higher subjective restfulness of sleep. Gender differences in sleep quality are frequently reported in current research, however, the nature and direction of these differences vary across studies (97–99). Hrozanova et al. (100) suggest that observed gender differences in subjective and objective sleep parameters may be influenced by the menstrual cycle in female athletes. Despite these findings, women remain underrepresented in sports science research, and the impact of the menstrual cycle is often overlooked or inadequately considered (101). Further research is urgently needed to better understand these gender-specific influences and to develop tailored approaches for care, prevention, and optimization of sleep-related behaviors in athletes.

Overall, the findings of the present study underscore the complexity and multidimensional nature of sleep quality, for which no universally accepted definition or operationalization currently exists (13). Recent research suggests that objective sleep measurements and subjective assessments of sleep quality are not as strongly correlated as one might expect (102). In a systematic review, Kirschen et al. (103) report that while objective sleep quality is particularly relevant for performance in sports requiring speed, tactical strategy, and technical skills, there remains a lack of studies examining the relationship between subjective sleep quality and athletic performance. Similarly, in their systematic review and meta-analysis, Gwyther et al. (104) highlight distinct effects of sleep interventions on subjective and objective sleep parameters. Given these findings, it may be beneficial to move beyond the broad distinction between subjective and objective sleep quality. Instead, differentiating between qualitative and quantitative sleep parameters may provide a more nuanced understanding. Both dimensions can be assessed through subjective and objective methods. Subjective approaches include sleep diaries and standardized screening questionnaires to evaluate qualitative (e.g., restfulness, recovery-stress states) and quantitative (e.g., subjective TIB, TST, SOL) aspects. Objective methods include actigraphy for assessing quantitative parameters and polysomnography for qualitative parameters, particularly regarding sleep architecture and the duration of specific sleep stages. Future research may benefit from shifting its focus from a general concept of sleep quality to a more comprehensive assessment of sleep that integrates these four dimensions.

### 4.1 Limitations

Despite its many strengths, this study has several limitations. First, the relatively small sample size and the short data collection period of only two weeks may have reduced the statistical power of the analyses. This is a common challenge in research involving elite athletes, as many are concerned that participation in studies may interfere with their training routines and performance. Moreover, elite athletes represent a rare and highly exclusive group, making the recruitment of larger samples particularly difficult. As such, while the sample size may appear small, it reflects the practical constraints of working with such a specialized population, where larger sample sizes are often not feasible. To mitigate this limitation, the study followed the recommendations of McNeish (73) for parameter estimation in multilevel modelling with small samples, and within-person reliabilities for the level-1 psychometric questionnaires were assessed before conducting statistical analyses. However, it should be noted that power analysis in the context of multilevel models remains challenging, especially when small samples and limited time points are involved. Conventional tools such as G*Power are not suitable for these types of models, as they do not account for the hierarchical structure of the data, which can lead to misestimations of required sample sizes. It is important to note that this study is observational in nature and aimed to capture the real-world sports routines of athletes. The primary goal was to identify practically relevant effects within the context of everyday athletic performance, rather than to detect large experimental effects. Consequently, it is possible that small effects were not detected with the current sample size. However, this limitation may also suggest that such effects are unlikely to be of significant practical relevance for the athletes in question, given the highly specific context of elite-level sport. Future studies with larger samples and longer data collection periods would benefit from examining these effects more robustly, enabling clearer insights into how interindividual variability and intraindividual fluctuations interact over time.

In addition to the limitations mentioned above, it is important to note that the sample consisted exclusively of judokas. At first glance, this limits the generalizability of the findings to other sports, as the specific demands and characteristics of judo may not fully reflect those of athletes in different disciplines. This is particularly true in more specialized or sport-specific contexts. However, since the majority of the data collection focused on sport-unspecific measures, such as psychological factors and recovery metrics, it is likely that the findings can be generalized to athletes in similar high-performance settings, even if they practice different sports. That being said, caution should be exercised when applying these results to athletes from other sports or different competitive levels. Therefore, future research should aim to include athletes from a wider range of sports and performance levels to further explore the applicability and robustness of the findings across diverse athletic populations.

Lastly, as this was an observational study, no active manipulation of variables was conducted, limiting the ability to draw causal conclusions. This limitation is particularly relevant for variables collected in the evening protocols. Although daily mindfulness was assessed retrospectively for the previous day, it remains possible that the observed relationship was bidirectional. That is, rather than higher daily mindfulness predicting better evening recovery scores, athletes may have retrospectively rated their mindfulness as higher on evenings when they experienced greater recovery. Consequently, the constructs identified as potential intervention targets require further investigation in future experimental studies.

## 5 Conclusion

The present study, utilizing a close monitoring design, demonstrates that both trait and daily mindfulness may be significant factors influencing sleep in elite athletes. Specifically, mindfulness appears to play a crucial role in subjective restfulness and in both morning and evening recovery-stress states. Furthermore, mindfulness affects sleep latencies at both the individual and interindividual levels. These findings are of particular relevance to populations experiencing sleep-related issues, as mindfulness interventions offer a relatively low-cost, non-invasive, and acute intervention option. Based on the present findings, acting with awareness should be considered a key aspect in the development and implementation of such interventions, with informal practice incorporated where feasible. To further elucidate the mechanisms underlying these effects, future research should include intervention studies that explore potential mediating factors, such as rumination or cognitive pre-sleep arousal. Finally, the results suggest that future research should conceptualize sleep as a multidimensional construct, considering both quantitative and qualitative parameters that can be assessed subjectively and objectively.

## Data Availability

The raw data supporting the conclusions of this article will be made available by the authors, without undue reservation.
