# Supplementary material for: Effect of daily mindfulness fluctuations on sleep and recovery-stress states in elite level judoka: an observational study
Source: Front Sports Act Living. 2025 Apr 24;7:1583058. doi: 10.3389/fspor.2025.1583058 (PMC12058686; doi:10.3389/fspor.2025.1583058)
Supplement: Supplementary file 1 [file Table1.docx]

**Supplementary Material**

**Supplementary Material A**

Parameter set for the multilevel analyses for objective quantitative sleep parameters.

| **Outcome** | ***B*** | ***SE*** | ***F*** | **(df)** | ***p*** |
| --- | --- | --- | --- | --- | --- |
| **O-TIB** | | | | | |
| Intercept | 478.401 | 9.712 | 2426.230 | (1, 49.581) | <.001 |
| MSMQ-1 | -2.789 | 4.303 | 0.420 | (1, 153.093) | .518 |
| MSMQ-2 | 7.512 | 4.388 | 2.931 | (1, 190.595) | .089 |
| MSMQ-3 | -4.178 | 6.171 | 0.458 | (1, 187.389) | .499 |
| Sessions | 2.085 | 7.240 | 0.083 | (1, 197.869) | .774 |
| RPE | -7.635 | 2.598 | 8.636 | (1, 199.931) | .004 |
| Recovery | -1.129 | 5.830 | 0.038 | (1, 195.663) | .847 |
| MAAS | -0.861 | 7.417 | 0.013 | (1, 23.849) | .909 |
| P-Nap | -8.473 | 9.973 | 0.722 | (1, 219.058) | .396 |
| O-Nap | -20.352 | 12.357 | 2.713 | (1, 222.937) | .101 |
| SC-Nap | -18.701 | 16.735 | 1.249 | (1, 166.182) | .265 |
| Gender | 56.212 | 10.371 | 29.380 | (1, 27.103) | <.001 |
| TC-E | 2.201 | 8.707 | 0.064 | (1, 151.282) | .801 |
| **O-TST** | | | | | |
| Intercept | 394.774 | 11.828 | 1114.014 | (1, 39.536) | <.001 |
| MSMQ-1 | 1.682 | 3.618 | 0.216 | (1, 148.619) | .643 |
| MSMQ-2 | 3.931 | 3.702 | 1.128 | (1, 184.213) | .290 |
| MSMQ-3 | -1.284 | 5.225 | 0.060 | (1, 177.933) | .806 |
| Sessions | -5.842 | 6.119 | 0.911 | (1, 190.938) | .341 |
| RPE | -6.880 | 2.207 | 9.723 | (1, 193.029) | .002 |
| Recovery | 6.597 | 4.944 | 1.781 | (1, 185.883) | .184 |
| MAAS | 4.538 | 10.318 | 0.193 | (1, 26.162) | .664 |
| P-Nap | -26.943 | 8.657 | 9.687 | (1, 209.721) | .002 |
| O-Nap | -21.200 | 10.887 | 3.792 | (1, 221.364) | .053 |
| SC-Nap | -23.720 | 15.118 | 2.462 | (1, 182.356) | .118 |
| Gender | 46.035 | 14.096 | 10.666 | (1, 27.906) | .003 |
| TC-E | 6.694 | 7.418 | 0.814 | (1, 147.933) | .368 |
| **O-SE** | | | | | |
| Intercept | 82.458 | 1.544 | 2853.649 | (1, 38.911) | <.001 |
| MSMQ-1 | 0.503 | 0.430 | 1.369 | (1, 166.729) | .244 |
| MSMQ-2 | 0.035 | 0.420 | 0.007 | (1, 201.612) | .934 |
| MSMQ-3 | 0.101 | 0.598 | 0.028 | (1, 200.345) | .866 |
| Sessions | -1.526 | 0.684 | 4.973 | (1, 204.723) | .027 |
| RPE | -0.099 | 0.249 | 0.159 | (1, 206.163) | .690 |
| Recovery | 1.323 | 0.556 | 5.667 | (1, 205.383) | .018 |
| MAAS | 0.936 | 1.374 | 0.464 | (1, 26.977) | .502 |
| P-Nap | -2.963 | 0.963 | 9.461 | (1, 210.981) | .002 |
| O-Nap | 0.111 | 1.188 | 0.009 | (1, 200.958) | .925 |
| SC-Nap | -0.343 | 1.770 | 0.038 | (1, 215.543) | .847 |
| Gender | -0.023 | 1.868 | 0.000 | (1, 28.484) | .990 |
| TC-E | 0.780 | 0.899 | 0.753 | (1, 132.748) | .387 |
| **O-SOL** |  |  |  |  |  |
| Intercept | 7.222 | 2.159 | 11.184 | (1, 40.280) | .002 |
| MSMQ-1 | 1.254 | 0.842 | 2.217 | (1, 156.805) | .139 |
| MSMQ-2 | 1.573 | 0.834 | 3.552 | (1, 193.537) | .061 |
| MSMQ-3 | -1.486 | 1.182 | 1.579 | (1, 193.954) | .210 |
| Sessions | 4.185 | 1.363 | 9.427 | (1, 202.399) | .002 |
| RPE | 0.270 | 0.493 | 0.298 | (1, 202.292) | .585 |
| Recovery | -1.851 | 1.105 | 2.806 | (1, 205.171) | .095 |
| MAAS | -3.920 | 1.740 | 5.075 | (1, 20.599) | .035 |
| P-Nap | 1.909 | 1.896 | 1.013 | (1, 222.423) | .315 |
| O-Nap | 2.420 | 2.336 | 1.073 | (1, 221.116) | .301 |
| SC-Nap | 11.671 | 3.349 | 12.148 | (1, 207.085) | <.001 |
| Gender | 2.180 | 2.404 | 0.822 | (1, 22.736) | .374 |
| TC-E | 1.210 | 1.733 | 0.487 | (1, 140.413) | .486 |
| **O-WASO** | | | | | |
| Intercept | 75.280 | 8.371 | 80.865 | (1, 42.863) | <.001 |
| MSMQ-1 | -5.430 | 2.643 | 4.221 | (1, 174.085) | .041 |
| MSMQ-2 | 1.137 | 2.542 | 0.200 | (1, 204.795) | .655 |
| MSMQ-3 | -0.571 | 3.621 | 0.025 | (1, 205.627) | .875 |
| Sessions | 4.810 | 4.099 | 1.377 | (1, 200.238) | .242 |
| RPE | -1.757 | 1.502 | 1.367 | (1, 208.274) | .244 |
| Recovery | -6.067 | 3.330 | 3.319 | (1, 200.607) | .070 |
| MAAS | -1.803 | 7.216 | 0.062 | (1, 27.261) | .805 |
| P-Nap | 13.125 | 5.747 | 5.216 | (1, 207.357) | .023 |
| O-Nap | -6.780 | 7.021 | 0.932 | (1, 191.476) | .335 |
| SC-Nap | -10.882 | 10.618 | 1.050 | (1, 222.306) | .307 |
| Gender | 9.915 | 9.855 | 1.012 | (1, 29.110) | .323 |
| TC-E | -5.115 | 5.579 | 0.840 | (1, 123.904) | .361 |

*Notes: Dependent variables: O-TIB = Objective Time in Bed, O-TST = Objective Total Sleep Time, O-SE = Objective Sleep Efficiency, O-SOL = Objective Sleep Onset Latency, O-WASO = Objective Wake After Sleep Onset; Independent variables: MSMQ-1 = Acting with Awareness, MSMQ-2 = Non-judgemental Acceptance, MSMQ-3 = Present-moment Attention, Sessions = Number of training sessions on the previous day, RPE = Average intensity of the training sessions, MAAS =* *Mindful Attention Awareness Scale, P-Nap = Completion of a power nap on the previous day (binary), O-Nap = Completion of another nap on the previous day (binary); SC-Nap = Completion of full sleep cycle nap an the previous day (binary); Gender = Gender of the participant (binary: 0 = male, 1 = female), TC-E = Training Camp Environment (binary: 0 = home training, 1 = training camp).*
