# Supplementary material for: Effect of daily mindfulness fluctuations on sleep and recovery-stress states in elite level judoka: an observational study
Source: Front Sports Act Living. 2025 Apr 24;7:1583058. doi: 10.3389/fspor.2025.1583058 (PMC12058686; doi:10.3389/fspor.2025.1583058)
Supplement: Supplementary file 2 [file Table2.docx]

**Supplementary Material**

**Supplementary Material B**

Parameter set for the multilevel analyses for subjective quantitative sleep parameters.

| **Outcome** | ***B*** | ***SE*** | ***F*** | **(df)** | ***p*** |
| --- | --- | --- | --- | --- | --- |
| **S-TIB** | | | | | |
| Intercept | 502.886 | 8.619 | 3404.584 | (1, 51.055) | <.001 |
| MSMQ-1 | 8.836 | 5.349 | 2.729 | (1, 197.896) | .100 |
| MSMQ-2 | -3.177 | 5.102 | 0.388 | (1, 224.624) | .534 |
| MSMQ-3 | -6.756 | 7.097 | 0.906 | (1, 239.190) | .342 |
| Sessions | 2.204 | 7.881 | 0.078 | (1, 231.607) | .780 |
| RPE | -8.862 | 2.904 | 9.310 | (1, 226.859) | .003 |
| Recovery | -1.169 | 6.734 | 0.030 | (1, 234.107) | .862 |
| MAAS | -5.104 | 6.842 | 0.557 | (1, 24.349) | .463 |
| P-Nap | -13.607 | 11.123 | 1.497 | (1, 246.343) | .222 |
| O-Nap | -16.085 | 13.169 | 1.492 | (1, 242.294) | .223 |
| SC-Nap | 0.464 | 17.951 | 0.001 | (1, 212.406) | .979 |
| Gender | 35.612 | 9.658 | 13.596 | (1, 27.947) | <.001 |
| TC-E | 6.767 | 9.850 | 0.472 | (1, 123.748) | .493 |
| **S-TST** | | | | | |
| Intercept | 458.785 | 9.161 | 2508.005 | (1, 41.476) | <.001 |
| MSMQ-1 | 12,.818 | 5.125 | 6.256 | (1, 175.459) | .013 |
| MSMQ-2 | -0.484 | 4.966 | 0.009 | (1, 207.990) | .923 |
| MSMQ-3 | -5.442 | 6.944 | 0.614 | (1, 226.696) | .434 |
| Sessions | -6.699 | 7.633 | 0.770 | (1, 225.105) | .381 |
| RPE | -9.631 | 2.820 | 11.661 | (1, 213.854) | <.001 |
| Recovery | 9.002 | 6.593 | 1.864 | (1, 223.063) | .173 |
| MAAS | 1.954 | 7.597 | 0.066 | (1, 21.538) | .799 |
| P-Nap | -25.829 | 11.059 | 5.455 | (1, 252.294) | .020 |
| O-Nap | -28.312 | 13.152 | 4.634 | (1, 248.902) | .032 |
| SC-Nap | 2.044 | 18.084 | 0.013 | (1, 214.729) | .910 |
| Gender | 37.396 | 10.637 | 12.360 | (1, 24.264) | .002 |
| TC-E | 1.814 | 9.347 | 0.038 | (1, 107.848) | .846 |
| **S-SE** | | | | | |
| Intercept | 91.646 | 1.409 | 4228.611 | (1, 49.862) | <.001 |
| MSMQ-1 | 1.058 | 0.971 | 1.188 | (1, 200.660) | .277 |
| MSMQ-2 | 0.297 | 0.930 | 0.102 | (1, 219.268) | .750 |
| MSMQ-3 | 0.311 | 1.292 | 0.058 | (1, 239.096) | .810 |
| Sessions | -1.847 | 1.443 | 1.638 | (1, 227.364) | .202 |
| RPE | -0.394 | 0.532 | 0.550 | (1, 221.133) | .459 |
| Recovery | 1.881 | 1.230 | 2.338 | (1, 230.233) | .128 |
| MAAS | 1.432 | 1.054 | 1.847 | (1, 20.019) | .189 |
| P-Nap | -2.851 | 2.002 | 2.027 | (1, 232.887) | .156 |
| O-Nap | -2.780 | 2.371 | 1.374 | (1, 230.021) | .242 |
| SC-Nap | 0.037 | 3.240 | 0.000 | (1, 209.144) | .991 |
| Gender | 1.914 | 1.511 | 1.606 | (1, 23.696) | .217 |
| TC-E | -1.421 | 1.766 | 0.647 | (1, 146.084) | .422 |
| **S-SOL** |  |  |  |  |  |
| Intercept | 10.985 | 3.389 | 10.504 | (1, 42.516) | .002 |
| MSMQ-1 | 1.004 | 1.772 | 0.321 | (1, 196.192) | .571 |
| MSMQ-2 | -0.151 | 1.697 | 0.008 | (1, 223.243) | .929 |
| MSMQ-3 | -5.580 | 2.379 | 5.501 | (1, 235.259) | .020 |
| Sessions | 4.216 | 2.592 | 2.646 | (1, 229.642) | .105 |
| RPE | 1.865 | 0.959 | 3.786 | (1, 225.828) | .053 |
| Recovery | -4.814 | 2.241 | 4.614 | (1, 230.736) | .033 |
| MAAS | 4.243 | 2.886 | 2.162 | (1, 23.815) | .155 |
| P-Nap | 1.853 | 3.768 | 0.242 | (1, 248.681) | .623 |
| O-Nap | 13.688 | 4.469 | 9.380 | (1, 243.053) | .002 |
| SC-Nap | 10.902 | 6.182 | 3.110 | (1, 233.392) | .079 |
| Gender | 5.404 | 4.014 | 1.813 | (1, 26.233) | .190 |
| TC-E | 6.198 | 3.308 | 3.512 | (1, 125.719) | .063 |

*Notes: Dependent variables: S-TIB = Subjective Time in Bed, S-TST = Subjective Total Sleep Time, S-SE = Subjective Sleep Efficiency, S-SOL = Subjective Sleep Onset Latency; Independent variables: MSMQ-1 = Acting with Awareness, MSMQ-2 = Non-judgemental Acceptance, MSMQ-3 = Present-moment Attention, Sessions = Number of training sessions on the previous day, RPE = Average intensity of the training sessions, MAAS =* *Mindful Attention Awareness Scale, P-Nap = Completion of a power nap on the previous day (binary), O-Nap = Completion of another nap on the previous day (binary); SC-Nap = Completion of full sleep cycle nap an the previous day (binary); Gender = Gender of the participant (binary: 0 = male, 1 = female), TC-E = Training Camp Environment (binary: 0 = home training, 1 = training camp).*
