# Supplementary material for: Effect of daily mindfulness fluctuations on sleep and recovery-stress states in elite level judoka: an observational study
Source: Front Sports Act Living. 2025 Apr 24;7:1583058. doi: 10.3389/fspor.2025.1583058 (PMC12058686; doi:10.3389/fspor.2025.1583058)
Supplement: Supplementary file 3 [file Table3.docx]

**Supplementary Material**

**Supplementary material C**

Parameter set for the multilevel analyses for subjective morning parameters.

| **Outcome** | ***B*** | ***SE*** | ***F*** | **(df)** | ***p*** |
| --- | --- | --- | --- | --- | --- |
| **S-RF** | | | | | |
| Intercept | 2.330 | 0.100 | 538.002 | (1, 52.603) | <.001 |
| MSMQ-1 | -0.209 | 0.062 | 11.499 | (1, 221.744) | <.001 |
| MSMQ-2 | 0.070 | 0.058 | 1.459 | (1, 236.762) | .228 |
| MSMQ-3 | -0.030 | 0.080 | 0.139 | (1, 242.377) | .710 |
| Sessions | 0.041 | 0.087 | 0.224 | (1, 226.923) | .636 |
| RPE | 0.068 | 0.033 | 4.330 | (1, 235.514) | .039 |
| Recovery | -0.022 | 0.075 | 0.082 | (1, 228.097) | .775 |
| MAAS | -0.198 | 0.079 | 6.236 | (1, 24.784) | .020 |
| P-Nap | 0.468 | 0.123 | 14.538 | (1, 231.611) | <.001 |
| O-Nap | 0.346 | 0.145 | 5.681 | (1, 226.175) | .018 |
| SC-Nap | 0.263 | 0.207 | 1.626 | (1, 239.512) | .204 |
| Gender | -0.334 | 0.111 | 9.051 | (1, 27.607) | .006 |
| TC-E | 0.174 | 0.120 | 2.105 | (1, 119.382) | .149 |
| **Physical Performance Capability** | | | | | |
| Intercept | 3.777 | 0.165 | 522.142 | (1, 38.226) | <.001 |
| MSMQ-1 | 0.188 | 0.074 | 6.466 | (1, 205.263) | .012 |
| MSMQ-2 | -0.100 | 0.070 | 2.011 | (1, 229.779) | .158 |
| MSMQ-3 | -0.017 | 0.098 | 0.029 | (1, 234.873) | .865 |
| Sessions | -0.232 | 0.106 | 4.844 | (1, 227.395) | .029 |
| RPE | -0.135 | 0.040 | 11.673 | (1, 230.780) | <.001 |
| Recovery | -0.045 | 0.092 | 0.244 | (1, 227.408) | .622 |
| MAAS | 0.084 | 0.147 | 0.324 | (1, 24.840) | .574 |
| P-Nap | -0.284 | 0.155 | 3.378 | (1, 228.893) | .067 |
| O-Nap | -0.206 | 0.183 | 1.263 | (1, 219.893) | .262 |
| SC-Nap | -0.273 | 0.262 | 1.086 | (1, 249.698) | .298 |
| Gender | 0.143 | 0.202 | 0.497 | (1, 26.515) | .487 |
| TC-E | -0.545 | 0.143 | 14.482 | (1, 108.106) | <.001 |
| **Mental Performance Capability** | | | | | |
| Intercept | 4.229 | 0.220 | 371.085 | (1, 36.143) | <.001 |
| MSMQ-1 | 0.170 | 0.082 | 4.322 | (1, 193.527) | .039 |
| MSMQ-2 | 0.029 | 0.079 | 0.136 | (1, 222.798) | .712 |
| MSMQ-3 | 0.084 | 0.110 | 0.584 | (1, 231.284) | .446 |
| Sessions | -0.364 | 0.119 | 9.360 | (1, 230.045) | .002 |
| RPE | -0.150 | 0.044 | 11.491 | (1, 226.288) | <.001 |
| Recovery | 0.111 | 0.104 | 1.152 | (1, 231.026) | .284 |
| MAAS | 0.258 | 0.203 | 1.605 | (1, 26.829) | .216 |
| P-Nap | -0.409 | 0.177 | 5.321 | (1, 237.868) | .022 |
| O-Nap | -0.298 | 0.210 | 2.006 | (1, 231.616) | .158 |
| SC-Nap | -0.410 | 0.298 | 1.892 | (1, 241.223) | .170 |
| Gender | 0.001 | 0.278 | 0.000 | (1, 28.074) | .997 |
| TC-E | -0.521 | 0.154 | 11.393 | (1, 114.926) | .001 |
| **Emotional Balance** | | | | | |
| Intercept | 4.222 | 0.214 | 388.326 | (1, 36.186) | <.001 |
| MSMQ-1 | 0.068 | 0.080 | 0.730 | (1, 196.504) | .394 |
| MSMQ-2 | 0.055 | 0.076 | 0.527 | (1, 225.413) | .469 |
| MSMQ-3 | 0.127 | 0.106 | 1.421 | (1, 232.516) | .234 |
| Sessions | -0.326 | 0.115 | 8.027 | (1, 230.172) | .005 |
| RPE | -0.116 | 0.043 | 7.314 | (1, 228.100) | .007 |
| Recovery | 0.142 | 0.100 | 1.996 | (1, 230.967) | .159 |
| MAAS | 0.260 | 0.198 | 1.715 | (1, 26.944) | .201 |
| P-Nap | -0.375 | 0.171 | 4.804 | (1, 233.731) | .029 |
| O-Nap | -0.025 | 0.203 | 0.015 | (1, 226.103) | .903 |
| SC-Nap | -0.377 | 0.289 | 1.699 | (1, 244.029) | .194 |
| Gender | 0.055 | 0.271 | 0.041 | (1, 28.160) | .841 |
| TC-E | -0.331 | 0.151 | 4.792 | (1, 112.052) | .031 |
| **Overall Recovery** | | | | | |
| Intercept | 3.594 | 0.179 | 403.119 | (1, 40.603) | <.001 |
| MSMQ-1 | 0.200 | 0.084 | 5.650 | (1, 218.030) | .018 |
| MSMQ-2 | -0.087 | 0.079 | 1.204 | (1, 232.081) | .274 |
| MSMQ-3 | -0.098 | 0.110 | 0.794 | (1, 234.579) | .374 |
| Sessions | -0.348 | 0.118 | 8.687 | (1, 224.523) | .004 |
| RPE | -0.233 | 0.045 | 27.358 | (1, 232.113) | <.001 |
| Recovery | 0.023 | 0.103 | 0.052 | (1, 222.683) | .820 |
| MAAS | 0.212 | 0.157 | 1.840 | (1, 24.931) | .187 |
| P-Nap | -0.310 | 0.171 | 3.281 | (1, 224.767) | .071 |
| O-Nap | -0.352 | 0.202 | 3.031 | (1, 216.994) | .083 |
| SC-Nap | 0.380 | 0.294 | 1.670 | (1, 254.108) | .197 |
| Gender | 0.006 | 0.216 | 0.001 | (1, 26.714) | .978 |
| TC-E | -0.376 | 0.167 | 5.075 | (1, 117.245) | .026 |
| **Muscular Stress** | | | | | |
| Intercept | 2.657 | 0.201 | 174.700 | (1, 38.027) | <.001 |
| MSMQ-1 | -0.066 | 0.086 | 0.592 | (1, 226.351) | .443 |
| MSMQ-2 | 0.089 | 0.079 | 1.248 | (1, 227.854) | .265 |
| MSMQ-3 | -0.058 | 0.110 | 0.282 | (1, 226.387) | .596 |
| Sessions | 0.519 | 0.118 | 19.508 | (1, 214.194) | <.001 |
| RPE | 0.210 | 0.045 | 21.947 | (1, 228.346) | <.001 |
| Recovery | 0.092 | 0.102 | 0.819 | (1, 207.919) | .367 |
| MAAS | -0.338 | 0.179 | 3.567 | (1, 24.651) | .071 |
| P-Nap | 0.234 | 0.170 | 1.891 | (1, 206.730) | .171 |
| O-Nap | 0.445 | 0.200 | 4.934 | (1, 199.557) | .027 |
| SC-Nap | 0.217 | 0.300 | 0.524 | (1, 252.436) | .470 |
| Gender | -0.327 | 0.246 | 1.768 | (1, 26.052) | .195 |
| TC-E | 0.315 | 0.179 | 3.113 | (1, 111.735) | .080 |
| **Lack of Activation** | | | | | |
| Intercept | 2.034 | 0.268 | 57.779 | (1, 35.108) | <.001 |
| MSMQ-1 | -0.190 | 0.091 | 4.350 | (1, 197.482) | .038 |
| MSMQ-2 | -0.018 | 0.086 | 0.044 | (1, 226.966) | .834 |
| MSMQ-3 | -0.095 | 0.121 | 0.628 | (1, 232.312) | .429 |
| Sessions | 0.269 | 0.130 | 4.261 | (1, 229.325) | .040 |
| RPE | 0.115 | 0.049 | 5.553 | (1, 228.914) | .019 |
| Recovery | 0.034 | 0.113 | 0.091 | (1, 229.253) | .764 |
| MAAS | -0.274 | 0.251 | 1.190 | (1, 27.387) | .285 |
| P-Nap | 0.049 | 0.193 | 0.065 | (1, 225.954) | .798 |
| O-Nap | -0.086 | 0.229 | 0.140 | (1, 216.813) | .709 |
| SC-Nap | 0.177 | 0.331 | 0.287 | (1, 246.357) | .593 |
| Gender | -0.163 | 0.343 | 0.227 | (1, 28.340) | .637 |
| TC-E | 0.575 | 0.175 | 10.781 | (1, 104.978) | .001 |
| **Negative Emotional State** | | | | | |
| Intercept | 1.813 | 0.232 | 61.072 | (1, 36.234) | <.001 |
| MSMQ-1 | -0.025 | 0.083 | 0.091 | (1, 221.215) | .763 |
| MSMQ-2 | -0.038 | 0.078 | 0.235 | (1, 230.045) | .628 |
| MSMQ-3 | -0.097 | 0.107 | 0.813 | (1, 228.698) | .368 |
| Sessions | 0.189 | 0.115 | 2.687 | (1, 219.611) | .103 |
| RPE | 0.073 | 0.044 | 2.796 | (1, 230.322) | .096 |
| Recovery | -0.087 | 0.100 | 0.763 | (1, 214.623) | .383 |
| MAAS | -0.139 | 0.215 | 0.418 | (1, 26.900) | .524 |
| P-Nap | 0.436 | 0.168 | 6.734 | (1, 209.317) | .010 |
| O-Nap | -0.073 | 0.198 | 0.135 | (1, 201.475) | .714 |
| SC-Nap | 0.567 | 0.296 | 3.659 | (1, 251.001) | .057 |
| Gender | -0.207 | 0.293 | 0.496 | (1, 27.934) | .487 |
| TC-E | -0.025 | 0.170 | 0.022 | (1, 112.470) | .883 |
| **Overall Stress** | | | | | |
| Intercept | 2.508 | 0.177 | 201.053 | (1, 47.847) | <.001 |
| MSMQ-1 | -0.098 | 0.084 | 1.340 | (1, 237.204) | .248 |
| MSMQ-2 | 0.088 | 0.077 | 1.312 | (1, 222.277) | .253 |
| MSMQ-3 | -0.041 | 0.105 | 0.151 | (1, 220.584) | .698 |
| Sessions | 0.419 | 0.112 | 13.959 | (1, 205.733) | <.001 |
| RPE | 0.230 | 0.043 | 28.310 | (1, 224.760) | <.001 |
| Recovery | -0.045 | 0.097 | 0.214 | (1, 195.896) | .644 |
| MAAS | -0.357 | 0.150 | 5.697 | (1, 26.670) | .024 |
| P-Nap | 0.179 | 0.160 | 1.250 | (1, 196.777) | .265 |
| O-Nap | 0.423 | 0.188 | 5.083 | (1, 192.168) | .025 |
| SC-Nap | -0.143 | 0.287 | 0.248 | (1, 246.223) | .619 |
| Gender | -0.089 | 0.207 | 0.187 | (1, 28.453) | .669 |
| TC-E | 0.279 | 0.187 | 2.232 | (1, 110.160) | .138 |

*Notes: Dependent variables: S-RF = Subjective Restfulness of Sleep (1 = very restful to 5 = not restful at all); Independent variables: MSMQ-1 = Acting with Awareness, MSMQ-2 = Non-judgemental Acceptance, MSMQ-3 = Present-moment Attention, Sessions = Number of training sessions on the previous day, RPE = Average intensity of the training sessions, MAAS =* *Mindful Attention Awareness Scale, P-Nap = Completion of a power nap on the previous day (binary), O-Nap = Completion of another nap on the previous day (binary); SC-Nap = Completion of full sleep cycle nap an the previous day (binary); Gender = Gender of the participant (binary: 0 = male, 1 = female), TC-E = Training Camp Environment (binary: 0 = home training, 1 = training camp).*
