# Supplementary material for: Effect of daily mindfulness fluctuations on sleep and recovery-stress states in elite level judoka: an observational study
Source: Front Sports Act Living. 2025 Apr 24;7:1583058. doi: 10.3389/fspor.2025.1583058 (PMC12058686; doi:10.3389/fspor.2025.1583058)
Supplement: Supplementary file 4 [file Table4.docx]

**Supplementary Material**

**Supplementary Material D**

Parameter set for the multilevel analyses for subjective evening parameters.

| **Outcome** | ***B*** | ***SE*** | ***F*** | **(df)** | ***p*** |
| --- | --- | --- | --- | --- | --- |
| **Physical Performance Capability** | | | | | |
| Intercept | 3.583 | 0.219 | 268.697 | (1, 36.993) | <.001 |
| MSMQ-1 | 0.268 | 0.079 | 11.505 | (1, 245.373) | <.001 |
| MSMQ-2 | -0.097 | 0.076 | 1.631 | (1, 236.202) | .203 |
| MSMQ-3 | 0.060 | 0.100 | 0.363 | (1, 227.698) | .547 |
| Sessions | -0.189 | 0.111 | 2.887 | (1, 226.563) | .091 |
| RPE | -0.081 | 0.042 | 3.764 | (1, 239.597) | .054 |
| Recovery | -0.040 | 0.092 | 0.187 | (1, 206.719) | .666 |
| MAAS | 0.078 | 0.198 | 0.153 | (1, 25.688) | .699 |
| P-Nap | 0.029 | 0.160 | 0.033 | (1, 216.434) | .856 |
| O-Nap | 0.129 | 0.185 | 0.489 | (1, 203.415) | .485 |
| SC-Nap | 0.212 | 0.287 | 0.544 | (1, 263.214) | .461 |
| Gender | -0.332 | 0.271 | 1.499 | (1, 26.592) | .232 |
| TC-E | -0.363 | 0.182 | 3.974 | (1, 123.690) | .048 |
| **Mental Performance Capability** | | | | | |
| Intercept | 3.933 | 0.254 | 240.446 | (1, 34.765) | <.001 |
| MSMQ-1 | 0.243 | 0.080 | 9.196 | (1, 233.009) | .003 |
| MSMQ-2 | -0.007 | 0.079 | 0.008 | (1, 244.318) | .927 |
| MSMQ-3 | 0.217 | 0.106 | 4.186 | (1, 244.781) | .042 |
| Sessions | -0.100 | 0.117 | 0.735 | (1, 244.496) | .392 |
| RPE | -0.043 | 0.044 | 0.974 | (1, 245.777) | .325 |
| Recovery | 0.206 | 0.099 | 4.312 | (1, 238.726) | .039 |
| MAAS | 0.369 | 0.239 | 2.389 | (1, 27.689) | .134 |
| P-Nap | 0.090 | 0.173 | 0.268 | (1, 245.010) | .605 |
| O-Nap | 0.228 | 0.203 | 1.258 | (1, 228.682) | .263 |
| SC-Nap | 0.561 | 0.297 | 3.569 | (1, 259.973) | .060 |
| Gender | -0.289 | 0.325 | 0.787 | (1, 28.378) | .382 |
| TC-E | -0.395 | 0.163 | 5.907 | (1, 122.260) | .017 |
| **Emotional Balance** | | | | | |
| Intercept | 4.037 | 0.198 | 417.264 | (1, 37.741) | <.001 |
| MSMQ-1 | 0.038 | 0.075 | 0.258 | (1, 242.790) | .612 |
| MSMQ-2 | 0.202 | 0.073 | 7.673 | (1, 245.407) | .006 |
| MSMQ-3 | 0.166 | 0.097 | 2.923 | (1, 239.463) | .089 |
| Sessions | -0.068 | 0.108 | 0.402 | (1, 239.248) | .527 |
| RPE | -0.055 | 0.040 | 1.887 | (1, 246.287) | .171 |
| Recovery | 0.092 | 0.090 | 1.043 | (1, 223.880) | .308 |
| MAAS | 0.197 | 0.180 | 1.200 | (1, 26.405) | .283 |
| P-Nap | -0.298 | 0.157 | 3.622 | (1, 232.444) | .058 |
| O-Nap | -0.094 | 0.182 | 0.264 | (1, 214.117) | .608 |
| SC-Nap | 0.372 | 0.273 | 1.855 | (1, 268.122) | .174 |
| Gender | 0.085 | 0.246 | 0.121 | (1, 27.422) | .731 |
| TC-E | 0.045 | 0.160 | 0.080 | (1, 112.709) | .777 |
| **Overall Recovery** | | | | | |
| Intercept | 3.305 | 0.188 | 308.178 | (1, 42.917) | <.001 |
| MSMQ-1 | 0.375 | 0.084 | 20.102 | (1, 248.165) | <.001 |
| MSMQ-2 | -0.149 | 0.081 | 3.361 | (1, 245.840) | .068 |
| MSMQ-3 | -0.050 | 0.108 | 0.214 | (1, 239.663) | .644 |
| Sessions | -0.343 | 0.120 | 8.152 | (1, 237.889) | .005 |
| RPE | -0.142 | 0.045 | 10.074 | (1, 246.782) | .002 |
| Recovery | -0.025 | 0.101 | 0.063 | (1, 223.762) | .802 |
| MAAS | 0.095 | 0.164 | 0.337 | (1, 26.474) | .567 |
| P-Nap | -0.294 | 0.173 | 2.876 | (1, 238.621) | .091 |
| O-Nap | -0.107 | 0.201 | 0.286 | (1, 223.615) | .594 |
| SC-Nap | 0.075 | 0.301 | 0.062 | (1, 270.743) | .803 |
| Gender | 0.021 | 0.226 | 0.009 | (1, 27.924) | .925 |
| TC-E | -0.460 | 0.180 | 6.572 | (1, 132.160) | .011 |
| **Muscular Stress** | | | | | |
| Intercept | 2.731 | 0.215 | 162.004 | (1, 41.282) | <.001 |
| MSMQ-1 | -0.223 | 0.087 | 6.487 | (1, 244.829) | .011 |
| MSMQ-2 | 0.013 | 0.084 | 0.023 | (1, 233.149) | .880 |
| MSMQ-3 | -0.162 | 0.111 | 2.139 | (1, 225.730) | .145 |
| Sessions | 0.285 | 0.123 | 5.383 | (1, 223.332) | .021 |
| RPE | 0.181 | 0.046 | 15.179 | (1, 237.554) | <.001 |
| Recovery | 0.006 | 0.101 | 0.003 | (1, 204.423) | .955 |
| MAAS | -0.205 | 0.188 | 1.190 | (1, 25.492) | .286 |
| P-Nap | -0.001 | 0.175 | 0.000 | (1, 218.413) | .994 |
| O-Nap | 0.291 | 0.202 | 2.083 | (1, 207.561) | .150 |
| SC-Nap | -0.044 | 0.315 | 0.019 | (1, 264.022) | .890 |
| Gender | -0.318 | 0.258 | 1.520 | (1, 26.661) | .228 |
| TC-E | 0.473 | 0.207 | 5.213 | (1, 134.654) | .024 |
| **Lack of Activation** | | | | | |
| Intercept | 1.802 | 0.245 | 54.197 | (1, 35.269) | <.001 |
| MSMQ-1 | -0.127 | 0.083 | 2.327 | (1, 220.170) | .129 |
| MSMQ-2 | -0.067 | 0.083 | 0.643 | (1, 236.652) | .423 |
| MSMQ-3 | -0.214 | 0.113 | 3.633 | (1, 246.993) | .058 |
| Sessions | -0.008 | 0.124 | 0.004 | (1, 242.169) | .947 |
| RPE | 0.054 | 0.046 | 1.364 | (1, 242.098) | .244 |
| Recovery | -0.185 | 0.106 | 3.040 | (1, 246.756) | .082 |
| MAAS | -0.327 | 0.228 | 2.053 | (1, 27.402) | .163 |
| P-Nap | 0.153 | 0.185 | 0.686 | (1, 258.069) | .408 |
| O-Nap | -0.126 | 0.219 | 0.331 | (1, 250.418) | .566 |
| SC-Nap | -0.329 | 0.310 | 1.132 | (1, 250.044) | .288 |
| Gender | 0.007 | 0.312 | 0.001 | (1, 28.247) | .981 |
| TC-E | 0.460 | 0.162 | 8.099 | (1, 127.393) | .005 |
| **Negative Emotional State** | | | | | |
| Intercept | 2.024 | 0.244 | 68.568 | (1, 36.591) | <.001 |
| MSMQ-1 | 0.160 | 0.089 | 3.197 | (1, 224.862) | .075 |
| MSMQ-2 | -0.293 | 0.089 | 10.961 | (1, 240.821) | .001 |
| MSMQ-3 | -0.216 | 0.119 | 3.271 | (1, 247.509) | .072 |
| Sessions | 0.029 | 0.132 | 0.050 | (1, 244.427) | .824 |
| RPE | 0.049 | 0.049 | 0.997 | (1, 244.324) | .319 |
| Recovery | -0.006 | 0.112 | 0.003 | (1, 245.260) | .960 |
| MAAS | -0.079 | 0.225 | 0.122 | (1, 27.128) | .730 |
| P-Nap | 0.021 | 0.196 | 0.011 | (1, 256.068) | .915 |
| O-Nap | -0.517 | 0.230 | 5.039 | (1, 242.814) | .026 |
| SC-Nap | -0.040 | 0.330 | 0.015 | (1, 256.213) | .903 |
| Gender | -0.224 | 0.308 | 0.528 | (1, 28.111) | .473 |
| TC-E | 0.119 | 0.177 | 0.451 | (1, 120.945) | .503 |
| **Overall Stress** | | | | | |
| Intercept | 2.804 | 0.219 | 163.575 | (1, 42.772) | <.001 |
| MSMQ-1 | -0.297 | 0.093 | 10.177 | (1, 248.947) | .002 |
| MSMQ-2 | 0.015 | 0.090 | 0.029 | (1, 241.857) | .864 |
| MSMQ-3 | -0.148 | 0.119 | 1.546 | (1, 233.596) | .215 |
| Sessions | 0.396 | 0.133 | 8.905 | (1, 231.917) | .003 |
| RPE | 0.196 | 0.050 | 15.564 | (1, 244.058) | <.001 |
| Recovery | 0.059 | 0.110 | 0.283 | (1, 213.328) | .595 |
| MAAS | -0.215 | 0.192 | 1.256 | (1, 26.714) | .272 |
| P-Nap | 0.340 | 0.190 | 3.183 | (1, 226.318) | .076 |
| O-Nap | -0.213 | 0.220 | 0.933 | (1, 211.552) | .335 |
| SC-Nap | -0.343 | 0.336 | 1.039 | (1, 269.785) | .309 |
| Gender | 0.208 | 0.264 | 0.617 | (1, 28.038) | .439 |
| TC-E | 0.186 | 0.208 | 0.805 | (1, 124.240) | .371 |

*Notes: Independent variables: MSMQ-1 = Acting with Awareness, MSMQ-2 = Non-judgemental Acceptance, MSMQ-3 = Present-moment Attention, Sessions = Number of training sessions on the previous day, RPE = Average intensity of the training sessions, MAAS =* *Mindful Attention Awareness Scale, P-Nap = Completion of a power nap on the previous day (binary), O-Nap = Completion of another nap on the previous day (binary); SC-Nap = Completion of full sleep cycle nap an the previous day (binary); Gender = Gender of the participant (binary: 0 = male, 1 = female), TC-E = Training Camp Environment (binary: 0 = home training, 1 = training camp).*
